# Soluble and Insoluble Lysates from the Human A53T Mutant α-Synuclein Transgenic Mouse Model Induces α-Synucleinopathy Independent of Injection Site

**DOI:** 10.3390/ijms26136254

**Published:** 2025-06-28

**Authors:** Justin Barnes, Scott C. Vermilyea, Joyce Meints, Héctor Martell-Martinez, Michael K. Lee

**Affiliations:** 1 Department of Neuroscience, Institute for Translational Neuroscience, University of Minnesota, Minneapolis, MN 55455, USA; bubberman13@gmail.com (J.B.); meint002@umn.edu (J.M.); martineh@umn.edu (H.M.-M.); 2 Aligning Science Across Parkinson’s (ASAP) Collaborative Research Network, Chevy Chase, MD 20815, USA; svermily@umn.edu

**Keywords:** alpha-synuclein, alpha-synucleinopathy, alpha-synuclein spreading, endoplasmic reticulum, oligomers, transgenic mouse

## Abstract

Pathological aggregation of α-synuclein (αS) is implicated in the pathogenesis of Parkinson’s disease (PD) and other α-synucleinopathies. The current view is that neuron-to-neuron spreading of αS pathology contributes to the progression of α-synucleinopathy. We used an A53T mutant human αS transgenic mouse model (*TgA53T*) to examine whether the site of pathogenic αS inoculation affects the pattern of neuropathology and whether soluble and insoluble fractions derived from crude pathogenic tissue lysates exhibit differential capacities to initiate αS pathology. To test whether the inoculation site impacts the ultimate spatial/temporal patterns of αS pathology, αS preformed fibrils (PFFs), or brain homogenates from *TgA53T* mice with α-synucleinopathy, were injected into the cortex/striatum, brainstem, or skeletal muscle. In all cases, inoculation of pathogenic αS induced end-stage motor dysfunction within ~100 days post-inoculation (dpi). Significantly, irrespective of the inoculation sites, the ultimate distribution of the αS pathology was like that seen in normally aged *TgA53T* mice at end-stage, indicating that the intrinsic neuronal vulnerability is a significant determinant in the induction of αS pathology, even when initiated by inoculation of pathogenic αS. Temporal analysis of brainstem-injected *TgA53T* mice show that initial αS pathology was seen by 30 days post-inoculation and inflammatory changes occur at later stages. In addition, we show that both highly soluble (S150) and insoluble (P150) fractions from end-stage *TgA53T* mice can seed de novo αS pathology in vivo. Moreover, the endoplasmic reticulum (ER)-enriched fraction from the TgA53T mice were highly pathogenic as the ER fraction induced αS pathology faster than other fractions when injected unilaterally into *TgA53T* mice. Our results suggest that multiple αS species from the brain can initiate the development of progressive αS pathology.

## 1. Introduction

Parkinson’s disease (PD) is a common late-onset, progressive neurodegenerative disorder characterized by the presence of intracellular aggregates of α-synuclein (αS) termed Lewy bodies (LBs) and neurites (LNs) [1]. While the etiology of PD is unknown in most cases, degenerating neuronal populations in PD exhibit αS abnormalities, indicating that the αS abnormalities are mechanistically linked to the pathogenesis of PD and other α-synucleinopathies [2,3].

The temporal analysis of αS pathology in human cases [4,5,6] indicates that αS pathology in the PD brain originates in several focal sites, particularly the dorsal motor nucleus of vagus (DMV) and the anterior olfactory nucleus. With the preclinical progression of the disease, αS pathology moves anteriorly in a topographically defined manner until reaching the cortex during the symptomatic stage. This staging led to the hypothesis that αS pathology moves via cell-to-cell transmission of amyloidogenic αS species that serves as a conformational template to seed pathology [4,5]. This hypothesis was also further supported by the presence of LBs in fetal dopamine neurons grafted into PD patients [7,8]. Cell-to-cell transmission of α-synucleinopathy has received formal experimental support from brain injection studies establishing αS spreads trans-synaptically in mice [9,10,11]. Currently, direct inoculation of αS preformed fibrils (PFFs) or tissue lysates/fractions containing αS aggregates can induce αS pathology when directly injected into the central nervous system (CNS) or peripherally [12]. However, most current studies use αS PFF derived from recombinant αS. Thus, it is unknown if the spatial–temporal patterns of αS pathology are comparable following the inoculation of various pathogenic αS species to various brain regions of αS Tg models.

To better understand the spreading of α-synucleinopathy, we directly compared spatial–temporal patterns of αS pathology following the inoculation of pathogenic αS species into different brain regions (cortex/striatum and dorsal brainstem) and skeletal muscle of A53T mutant human αS (*TgA53T*) transgenic mice. We show that the resulting pattern of pathology is independent of injections sites, indicating that specific neuronal populations are predisposed to the effects of toxic αS species in vivo. We also show that both the soluble and insoluble fractions from end-stage *TgA53T* mice can initiate pathology in younger *TgA53T* mice and endoplasmic reticulum (ER)-associated αS is more pathogenic than other fractions.

## 2. Results

### 2.1. Lysates from Symptomatic TgA53T Mice and αS PFF Induce α-Synucleinopathy with Similar End-Stage Distribution

**Figure 1:**
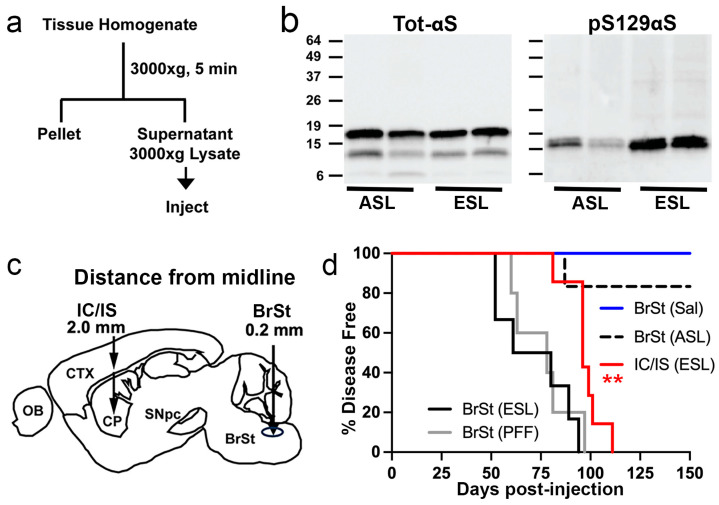
Injection of *TgA53T* brain-derived lysate into cortex/striatum or brainstem induces α-synucleinopathy and premature death in young *TgA53T* mice. (**a**) Schematic representation of the protocol used to generate the injected ESL and asymptomatic ASL. (**b**) Analysis of ASL and ESL for total αS (top) and pS129 αS levels. While similar levels of total αS are present in ASL and ESL, ESL contains more pS129 αS than ASL. (**c**) Diagrams depicting the stereotaxic injection sites for the IC/IS and BrSt injection models. Stereotaxic coordinates: IC/IS 2.0 mm lateral from the midline, +0.2 mm relative to bregma, and 0.8 and 2.6 mm deep from the dura; BrSt: 0.2 mm lateral from the midline, −7.34 mm from bregma, 3.75 mm deep from the dura. (**d**) Kaplan–Meier survival curve of mice IC/IS injected with ESL and BrSt-injected animals with αS PFF, ESL, ASL, and saline. Control animals receiving saline and ASL all survived disease free to 200 days post-injection (dpi), except one animal injected with ASL that developed disease and died 136 dpi. ** *p* < 0.01, IC/IS ESL vs BrSt ESL or BrSt PFF, Mantel–Cox log-rank test. All *n* = 6 except BrSt (PFF), *n* = 5. Abbreviations: α-synuclein, αS; asymptomatic lysate, ASL; end-stage lysate, ESL; intracortical/intrastriatal, IC/IS; brainstem, BrSt; preformed fibril, PFF.

Although the intracortical/intrastriatal (IC/IS) transmission model shows the induction and spread of αS pathology in TgA53T mice [9], studies show that injections of pathogenic αS lysates or preformed αS fibrils (PFFs) in multiple areas, including the skeletal muscle, can induce widespread αS pathology over time [13]. Thus, we examined whether the inoculation site impacts the onset and spread of αS pathology in TgA53T mice. Because brainstem (BrSt) neurons are affected early by αS pathology in both human PD [14] and in the TgA53T mouse model [15], we compared the disease produced by the IC/IS inoculation with the BrSt injections of αS PFF. We injected young, disease-free 3–6-month-old TgA53T mice (all from line G2-3 unless noted) with end-stage lysate (ESL or S3000, a crude 3000× *g* pooled lysate) from BrSt/Spinal Cord (SpC) of end-stage (ES)-affected TgA53T mice or lysates from disease-free (asymptomatic lysate, ASL) 5–6 months-old TgA53T mice (Figure 1a–c). Immunoblot analysis (Figure 1b) shows that both ASL and ESL contain a similar amount of total αS but the ESL contains a greater amount of pS129αS, consistent with pathology in ES TgA53T mice. Like that seen by Luk et al. [9], the mean of survival for bilateral IC/IS-injected mice was 98 ± 8.6 days post-inoculation (dpi) (Mean ± SD) (Figure 1d and Figure S1). Unilateral inoculation of ESL into the BrSt resulted in earlier onset of motor dysfunction with a mean survival of 71.3 ± 18.7 dpi (Figure 1d and Figure S1a), a significantly shorter disease time course than with IC/IS dual injections. Inoculation of αS PFF into BrSt leads to a mean survival time (77.4 ± 13.16 dpi, *n* = 5) that is like the ESL inoculation (Figure 1d and Figure S1a). All the mice injected with ASL or saline remained disease free at ~150 dpi (Figure 1d) except one ASL-inoculated animal who succumbed to disease and prematurely died at ~9 months of age, a time point where normal-aged TgA53T mice start to show disease [15]. Thus, it is likely that this animal developed disease independently of the injected lysate.

**Figure 2:**
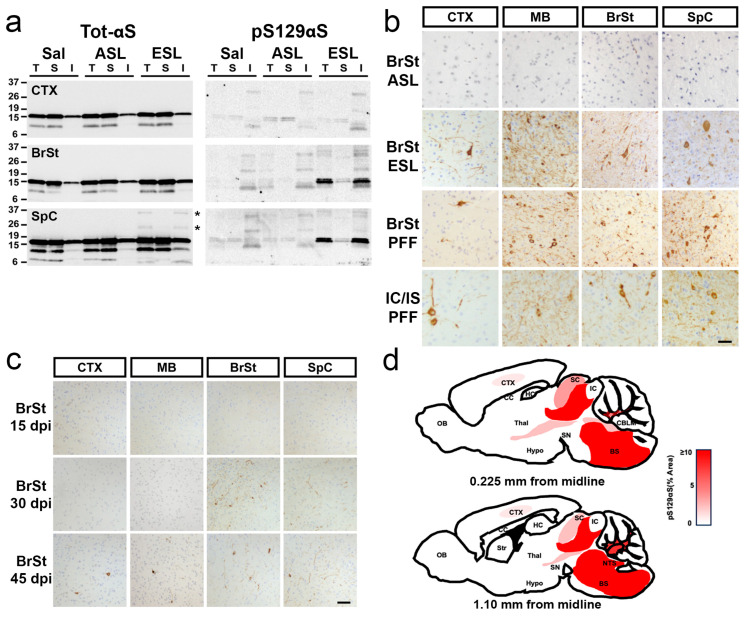
Aggregation of αS is induced by ESL in *TgA53T* mice with pathological distribution that is independent of injections site. (**a**) Accumulation of pathological HMW αS species (*) and pS129 αS in affected brain regions in detergent-insoluble fraction. Total lysates (T) from unaffected CTX and affected BrSt and SpC from saline, ASL, and ESL of BrSt-injected animals were separated into TX-soluble (S) and TX-insoluble (I) fractions. Detection of total αS (left) demonstrates the presence of HMW-insoluble αS species in SpC (*) ESL-inoculated mice. Immunoblots for pS129 αS clearly show a complete lack of pathological αS in ASL-injected animals and CTX of ESL-inoculated mice. Virtually all of the HMW αS species (*) and pS129 αS partition with the TX-insoluble fraction in BrSt and SpC. (**b**) Aggregated αS, marked by pS129 αS immunoreactivity, is abundant in animals injected with ESL or αS PFF. (**c**) Immunoreactivity for pS129 αS occurs between 15 and 30 days post-BrSt injection of ESL. (**d**) Diagrams depicting the distribution of αS pathology as observed by immunohistochemical analysis. Shades of red denote the relative abundance of αS inclusions (% Area covered by pS129 αS). Note that the sections are unilateral to the injection site even though pathology is detected bilaterally. Abbreviations: α-synuclein, αS; asymptomatic lysate, ASL; end-stage lysate, ESL; high molecular weight, HMW; cortex, CTX; brainstem, BrSt, BS; cerebellum, CBLM; corpus callosum, cc; hippocampus, HC; hypothalamus, Hypo; inferior and superior colliculus, IC, SC; nucleus tractus solitarius, NTS; olfactory bulb, OB; spinal cord, SpC; striatum, Str; substantia nigra, SN; thalamus, Thal; triton-X, TX; preformed fibril, PFF; saline, Sal. Scale bars: 50 µm.

Biochemical analysis of the ESL-inoculated animals confirms that the ESL, but not ASL, leads to αS pathology (Figure 2a). Analysis of Triton X100-detergent soluble and insoluble fractions reveals the accumulation of insoluble high molecular weight (HMW) species of αS (Figure 2a, asterisks) in the SpC of ESL-injected mice. In the ESL-injected mice, insoluble pS129αS accumulates in BrSt and SpC, corresponding to the regions most affected by αS pathology (Figure 2a). Consistent with lack of αS pathology, pS129αS does not accumulate in ASL- or saline-injected mice (Figure 2a). This pattern of αS pathology is equivalent to that seen with the ES TgA53T mice that normally develop α-synucleinopathy from aging [15].

Immunohistochemical detection of pS129αS demonstrates robust staining in multiple brain tissues from IC/IS- and BrSt-injected end-stage animals (Figure 2b). Consistent with the lack of motor phenotype, no pathological alterations are noted in ASL-inoculated mice or saline-injected animals (Figure 2b). Significantly, despite the differences in the site of inoculation (IC/IS vs BrSt), the overall patterns of pathology in the ES mice were comparable to each other with the most abundant pathology in the BrSt and SpC (Figure 2b), a pattern very similar to what is seen in the TgA53T mice that naturally develop αS pathology with aging [15]. However, the IC/IS-injected animals exhibit more pS129αS pathology in CTX compared to BrSt-injected animals (Figure S1b). To determine if components of lysates other than pathogenic αS could be responsible for the distribution of the pathology, we also examined animals that were BrSt-injected with αS PFF. Our results show that the pattern of pathology seen in the ESL-inoculated animals is very similar to animals injected with αS PFF into BrSt (Figure 2b and Figure S1c). Moreover, the pattern of αS pathology induced by BrSt injections is also very similar to the pathology achieved in this line of mice by IC/IS and peripheral, intramuscular (IM) inoculation of αS PFF [16,17]. Analysis of spinal cords from animals unilaterally injected with ESL (IC/IS) or αS-PFF (IM) confirm that αS pathology progresses to bilateral pathology (Figure S1d).

To determine if the prominent subcortical pathology is unique to the TgA53T(G2-3) line or occurs in another line of TgA53T, we performed IC/IS injections on TgA53T(H5), which express 50% less αS than TgA53T(G2-3) [18]. Because of lower transgene expression, injected TgA53T(H5) animals developed a progressive motor phenotype later (~150 dpi) than the TgA53T(G2-3) (~100 dpi) following IC/IS injections of αS PFF. Neuropathological analysis shows that, as with TgA53T(G2-3), αS pathology in ES mice was most abundant in the BrSt/SpC region (Figure S2). Significantly, the TgA53T(H5) animals also showed a more widespread distribution of αS pathology, including αS pathology in the hippocampus (Figure S2a). In summary, all animals inoculated with ESL or αS PFF showed the most severe BrSt and SpC αS pathology, regardless of the initial sites of injections.

Collectively, these results indicate that in TgA53T mice, the ES distribution of αS pathology is independent of the inoculation site and likely determined by factors other than local seeding by pathogenic αS. Our Tg models show the highest levels of total αS in the CTX [17] (Figure S2c). Thus, the relative lack of forebrain pathology is not because of insufficient αS expression in this area. However, because endogenous αS or βS could impact αS aggregation [19,20,21,22,23], we examined the expression of human αS along with levels of endogenous mouse αS and βS in various brain regions of TgA53T mice (Figure S2c). The results confirm that the transgene-derived HuαS is widely expressed in all brain regions. However, endogenous mouse αS and βS are completely absent from BrSt/SpC regions (Figure S2c), suggesting that the lack of endogenous αS and/or βS might contribute to the vulnerability of neurons to developing αS pathology in a TgA53T model.

While previous studies with the IC/IS injection model show that cortical and striatal αS pathology occurs prior to BrSt or SpC [9], we observe more prominent early subcortical pathology with the BrSt inoculation. Thus, following the BrSt inoculation, the appearance of pS129αS over time shows that initial αS pathology develops proximal to the inoculation site where αS pathology is seen in the pons by 30 dpi (Figure 2c). By 45 dpi, regions rostral and caudal to the BrSt inoculation sites exhibit αS pathology (Figure 2c). Thus, we propose that following BrSt injection of pathogenic αS, the pathology spreads in both rostral and caudal directions but the most prominent pathology occurs in subcortical regions (Figure 2d). Analysis of IC/IS-injected TgA53T(H5) animals at the intermediate stage (90 dpi) shows cortical pS129αS but the BrSt and SpC pS129αS pathology was more prominent (Figure S2b). Overall, the current results collectively show that regardless of the initial injection site, subcortical areas (e.g., BrSt, SpC) exhibit the earliest αS pathology and culminate in the most severe αS pathology in ES TgA53T mice.

### 2.2. Neuroinflammation Follows Onset of α-Synucleinopathy

**Figure 3:**
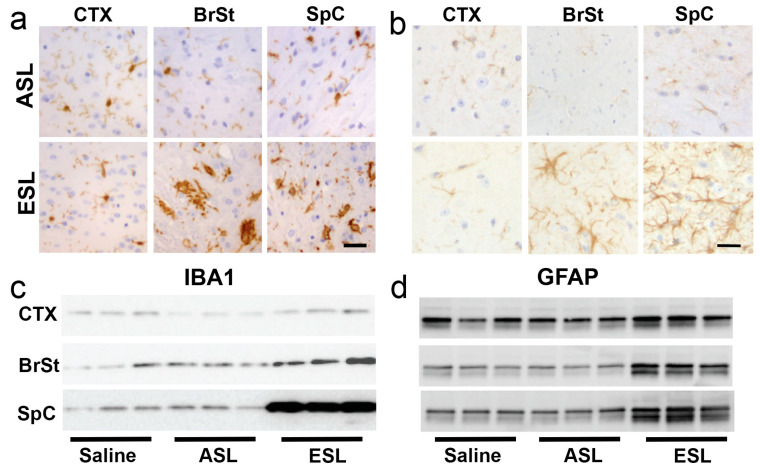
Neuroinflammation is increased in affected brain regions. (**a**,**b**) Tissue sections stained for Iba1 (**a**) and GFAP (**b**). BrSt and SpC from ESL-injected TgA53T mouse reveal abundant activated microglia and astrocytes. Areas lacking αS pathology, such as the CTX, as well as animals injected with ASL, are free of glial activation. (**c**,**d**) Biochemical analysis of whole-tissue lysates from CTX, BrSt, and SpC of saline-, ASL-, and ESL-injected mice show robust increases in Iba1 (**c**) and GFAP (**d**) in BrSt and SpC of ESL-injected mice. Quantitation of the blots is shown in Figure S3. Abbreviations: alpha synuclein, αS; brainstem, BrSt; spinal cord, SpC; end-stage lysate, ESL; cortex, CTX; asymptomatic lysate, ASL. Scale bars: 25 µm.

It has been proposed that inoculation with pathogenic αS could lead to early neuroinflammation that may promote further propagation of α-synucleinopathy [24]. Thus, we examined the spatial–temporal relationship between αS pathology and neuroinflammation. First, we confirmed that at ES, animals following BrSt injection exhibit coincident αS pathology and neuroinflammation. Tissue sections from BrSt-injected animals were stained for microglia (Iba1; Figure 3a) and astrocytes (GFAP; Figure 3b). Based on the morphology of the cells, no obvious neuroinflammatory changes are seen in the absence of significant αS pathology, such as in CTX (Figure 3a,b) and in animals injected with ASL (Figure 3a,b). However, in ES animals following ESL injection, areas with significant αS pathology (BrSt, SpC) exhibit abundant microglia and astrocytes with highly activated morphology. Immunoblotting for Iba1 and GFAP reveals no change in Ctx, but a significant increase in BrSt and SpC of ESL-injected mice at ES disease (Figure 3c,d and Figure S3).

**Figure 4:**
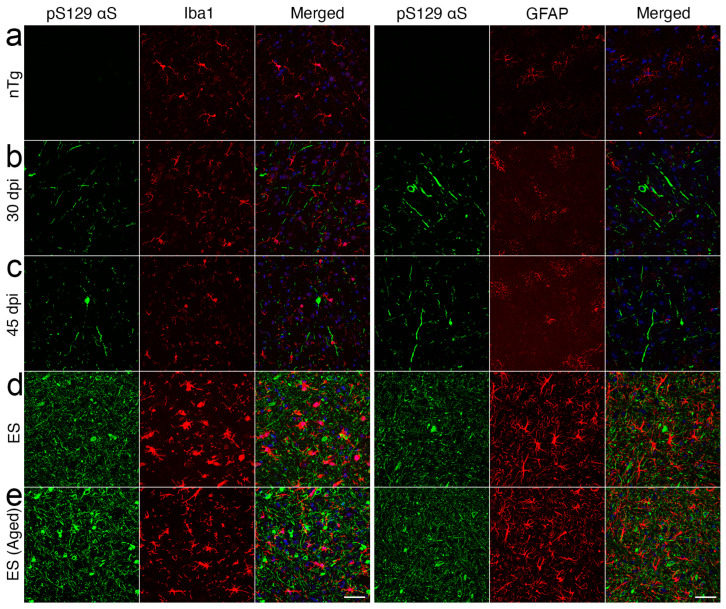
Neuroinflammation occurs after αS pathology in *TgA53T* mice. BrSt sections from control mice (**a**, ntg) and *TgA53T* mice injected with ESL into BrSt were analyzed at 30 dpi (**b**) 45 dpi (**c**), and ES (**d**, injected); (**e**, aged). The sections were stained for pS129 αS and Iba1 or GFAP. Aggregated αS (pS129 αS) is clearly seen in 30- and 45-dpi samples (**b**,**c**) but the overall morphology and density of Iba-1 and GFAP staining are comparable to nTg mice (**a**), indicating that the presence of pS129 αS is not accompanied by obvious glial activation. In contrast, there is a clear increase in Iba1 and GFAP staining with well-established pS129 αS pathology in ES animals (**d**,**e**). Abbreviations: alpha synuclein, αS; brainstem, BrSt; end-stage lysate, ESL; end-stage, ES; non transgenic, nTg. Scare bar: 50 μm.

To better determine the spatial–temporal relationship between the onset of microglial activation and αS pathology, the sections from BrSt-inoculated mice harvested at 15-, 30-, and 45-dpi that were evaluated for αS pathology (Figure 2c) were stained for microglia (Iba-1) and astrocytes (GFAP) (Figure S4). With BrSt inoculation, αS pathology is seen in the pons by 30 dpi (Figure 2c). However, there is no obvious signs of astrocytic or microglial activation at 30- or 45-dpi (Figure S4). Because αS pathology is still sparse at 45 dpi, we performed double immunofluorescence analysis of pS129αS with Iba1 or GFAP (Figure 4) to determine if the presence of αS pathology could be associated with local inflammatory changes. Double immunofluorescence analyses show that αS pathology in ES mice is associated with the nearby reactive astrocytes and microglia. However, such glial responses were not coincident with αS pathology at 30- or 45-dpi (Figure 4b,c). Similar analysis of early-stage mice following IM αS PFF injections shows that the initial onset of pS129αS pathology in SpC, occurring as early as 15–30 dpi, is not accompanied by obvious glial activation (Figure S5). While other A53T Tg models show αS pathology in astrocytes [25,26], we do not observe significant pS129αS in astrocytes in ES mice (Figure 4).

Collectively, our results indicate that glial activation, particularly activation of microglia, is only evident after substantial αS pathology is established. A similar pattern of initial αS pathology followed by neuroinflammation was reported with IM inoculation of the M83 line [27]. Thus, while microglia and astrocytes may modulate neuronal spreading of αS pathology or neuronal survival, our results indicate that it is unlikely that neuroinflammation is a significant factor in the initial onset of αS pathology in a TgA53T model of α-synucleinopathy.

### 2.3. Both Highly Soluble and Insoluble Fractions Induce αS Pathology

**Figure 5:**
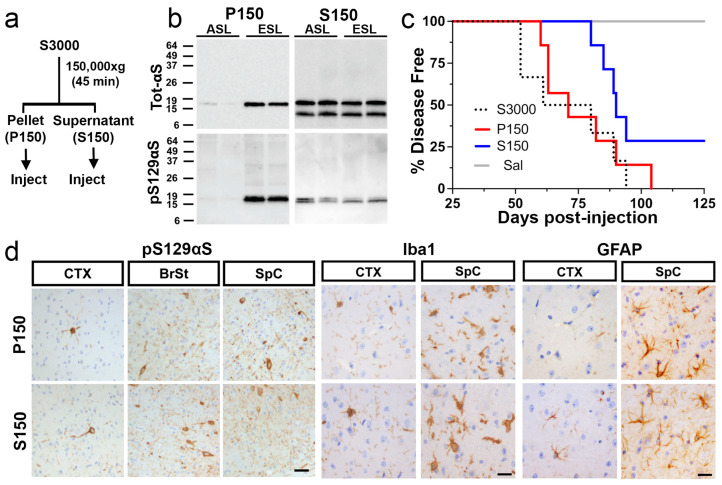
Both soluble and insoluble fractions from ESL induce α-synucleinopathy in *TgA53T* mice. (**a**) Flow diagram showing the separation of S3000 into soluble (S150) and insoluble (P150) fractions. (**b**) Biochemical analysis of S150 and P150 fractions derived from ASL and ESL. Equal amount of total protein was analyzed. P150 fractions from ASL contain very little αS while total αS and pS129 αS are abundant in P150 fractions from ESL. Despite the abundant total αS in S150, much lower pS129 αS is present in the S150. (**c**) Kaplan–Meier survival curve of mice injected with S150 and P150 into BrSt. Also shown in Figure 1d is the curve for the S3000-ESL-injected mice. Both S150 and P150 injections lead to motor dysfunction and reduced life span. S3000, *n* = 6; P150, *n* = 7; S150, *n* = 7; Sal, *n* = 6. (**d**) Neuropathological analysis of affected S150- and P150-injected animals shows expected distribution of αS pathology (pS129 αS) and corresponding neuroinflammation (Iba1 and GFAP). Abbreviations: end-stage lysate, ESL; asymptomatic lysate, ASL; α-synuclein, αS. Scale bars: 50 µm for pS129 αS, 25 µm for Iba1 and GFAP.

Because αS fibrils and disease-associated αS aggregates in TgA53T mice are detergent-insoluble [9,13,16,27], we tested whether the induction of αS pathology by the ESL is mediated by the insoluble αS species. The pathogenic S3000 ESL was centrifuged at 150,000× *g* to obtain highly soluble (supernatant, S150) and insoluble (pellet, P150) fractions (Figure 5a). Biochemical analyses of the fractions show that very little αS is found in P150 from asymptomatic animals (Figure 5b). Moreover, most of the pS129αS, representing the overt αS pathology, is highly enriched in the P150 fractions from the symptomatic mice (Figure 5b). In contrast, the amount of total αS is the same in the S150 fractions, regardless of the disease state of the animal (Figure 5b). Despite the high levels of total αS, the levels of pS129αS in S150 are lower than in the P150 with no differences between the disease state of the animal (Figure 5b). Side-by-side comparison and quantitative analysis of αS species in ESL (S3000), S150, and P150 show that, compared to ESL, pS129αS is highly enriched in the P150 fraction and the S150 fraction is low in pS129αS (Figure S6). Thus, we expected that αS pathology would be selectively induced by the P150-ESL fraction.

Following BrSt injections of P150 and S150 fractions, we were surprised to find that, regardless of the solubility of the inoculated material, inoculated TgA53T mice developed motor dysfunction leading to premature death with average lifespans of 75 ± 7 dpi for the P150 fraction and 88 ± 3 dpi for the S150 fraction (Figure 5c). While two out of seven subjects injected with S150 did not develop a disease phenotype by 150 dpi, there was no differences in the average lifespan (Figure 5c, *p* = 0.1397) of symptomatic animals injected with either S150 or P150.

Histological (Figure 5d) and biochemical (Figure S6) analyses of ES P150- and S150-injected animals demonstrate virtually indistinguishable αS pathology both in the spatial pattern and severity of αS aggregation (compare Figure 2, Figure 5 and Figure S6). Aggregation of αS occurred throughout the CNS but was most robust in the BrSt and SpC (Figure 5d). Biochemical analysis of brain tissue also reveals an accumulation of detergent-insoluble αS species in the insoluble fraction from BrSt and SpC (Figure S6a) and dramatic increases in pS129αS in both BrSt and SpC (Figure S6b), a pattern very similar to ESL-injected mice (Figure S7). Finally, based on immunostaining for Iba1 and GFAP, the distribution and intensity of neuroinflammation are similar in S150- and P150-BrSt-injected animals (Figure 5d), and are nearly identical to those observed in ESL-injected mice (see Figure 3). Thus, by three broad metrics- survival, immunohistochemical staining, and biochemistry- the α-synucleinopathy and disease induced by injection of all three end-stage lysates (S3000, S150, P150) are essentially identical.

Since S3000 ASL does not induce disease and there are no obvious differences between S150 from ASL and ESL by immunoblot analysis (Figure 5b), S150 from ESL may contain soluble pathogenic αS conformers that are SDS-labile. Thus, the incomplete penetrance of end-stage S150 to cause disease could be due to the possibility that toxic αS assemblies in S150 fractions are more prone to degradation following cellular uptake compared to more mature insoluble toxic αS species in P150 (Figure 5b). To test this hypothesis, we performed dot blot analysis of P150 and S150 for αS oligomers, using FILA1 [28] and the OC antibody [29] (Figure S8a,b). We previously showed that FILA1 can recognize both soluble and insoluble αS aggregates derived from the TgA53T model [30] and the OC antibody selectively recognizes mature fibrils [29]. Our results show that while levels of FILA1+ oligomers are similar between P150 and S150, the levels of OC+ oligomers are significantly more abundant in P150 (Figure S8a,b). We also subjected S150 and P150 to proteinase-K treatment as more compact mature aggregates should be more resistant to proteinase-K treatment. The results show that αS in P150 is more resistant to proteinase K proteolysis than αS in S150 (Figure S8c). Thus, we conclude that the presence of FILA1+ oligomers/aggregates in both S150 and P150 is pathogenic. Further, more labile FILA1+ oligomers in S150 may be responsible for the partial penetrance of S150 in inducing disease.

### 2.4. Microsomes from Symptomatic TgA53T Mice Induce Rapid α-Synucleinopathy

In the TgA53T model, α-synucleinopathy is associated with the accumulation of αS oligomers/aggregates in the endoplasmic reticulum (ER) and chronic ER stress that contributes to neurodegeneration [30,31]. Analyses of TgA53T mice inoculated with S3000 ESL show that αS pathology in these mice is also associated with signs of chronic ER stress (Figure S9), suggesting that ER stress occurs even when α-synucleinopathy is induced by exogenous inoculation of pathogenic αS. We recently showed that ER-enriched microsomes from TgA53T mice, containing αS aggregates, are highly toxic to cultured neurons and aggressively induce αS aggregates and cell death in cultured neurons [32]. Thus, we examined whether the microsome fraction from symptomatic TgA53T mice can induce pathology following BrSt injection.

**Figure 6:**
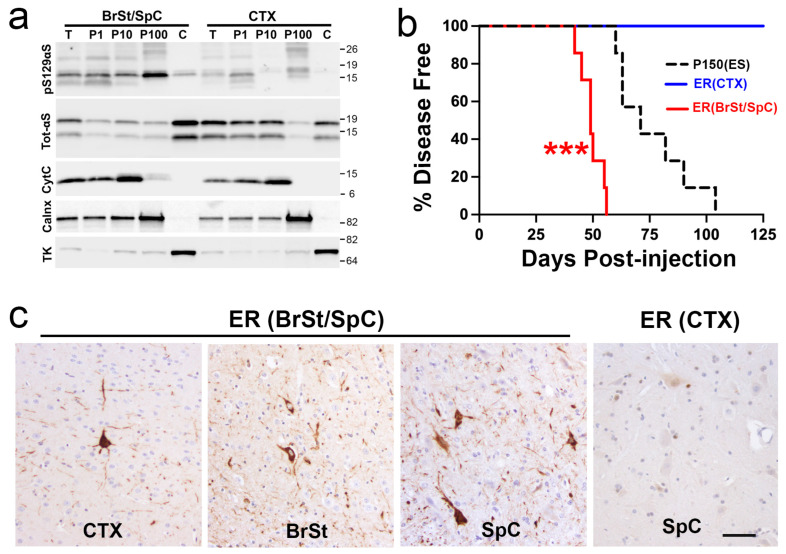
ER-enriched fraction from ES *TgA53T* mice, containing ER-associated αS, is highly pathogenic. (**a**) Subcellular fractions from BrSt/SpC and CTX were analyzed for pS129 αS, total αS, and organelle markers (Cytochrome C, CytC-mitochondria; Calnexin, Calnx-ER; Transketolase, KT-Cytosol). Calnexin-enriched ER fraction (P100) is relatively free of CytC and TK. pS129 αS is enriched in P100 from BrSt/SpC. (**b**) Kaplan–Meier survival curve of mice injected with P100 (ER) fractions from BrSt/SpC (BS/SC) or CTX. For reference, curve for P150-injected animals from Figure 4c is also shown. *** p < 0.001, ER (BrSt) vs. P150 (ES), Mantel–Cox log-rank test. P150 (ES), *n* = 7; ER (BrSt/SpC), *n* = 7; ER (CTX), *n* = 6. (**c**) Neuropathological analysis shows that ER (BS/SC) fraction induces pS129 αS pathology in *TgA53T* mice, particularly in BrSt and SpC. In contrast, ER (CTX) fraction does not cause pS129 pathology. Abbreviations: endoplasmic reticulum, ER; end-stage, ES; α-synuclein, αS; brainstem, BrSt/BS; spinal cord, SpC/SC; cortex, CTX. Scale bar: 50 µm.

Microsomes from pooled BrSt/SpC or CTX from the symptomatic TgA53T mice were used for BrSt injections of 3–4-month-old TgA53T mice. Analysis of the fractions shows that pS129αS is enriched in microsomes (P100 or ER) from BrSt/SpC, where very little pS129αS is seen in P100 from CTX (Figure 6a). Comparative analysis of total αS and pS129αS in various fractions from end-stage TgA53T mice indicates that the P100 fraction contains similar levels of total αS and pS129αS as the P150 fraction (Figure S9b). Analysis of organelle markers shows that the P100 fraction is also enriched in the ER marker (Grp78/BiP). Analysis of injected animals shows that P100 from symptomatic TgA53T mice develop progressive motor abnormalities by ~50 dpi while the animals inoculated with P100 from CTX did not show any disease phenotype (Figure 6b). Significantly, microsome inoculation leads to onset of motor symptoms much faster than any of the other fractions tested in this study (compare Figure 1d, Figure 5c and Figure 6b), indicating that microsome-associated αS oligomer/aggregates are highly pathogenic in vivo. Neuropathological analysis shows that the early motor deficits were indeed associated with significant αS pathology (Figure 6c) with the pattern seen with other αS fractions. Collectively, the results show that ER-associated αS oligomers/aggregates are highly pathogenic in vivo [30,32].

## 3. Discussion

In this report, we provide a comparative analysis of αS pathology induced by the inoculation of lysates from *TgA53T* mice. Specifically, we examined the induction of αS pathology as a function of different lysate solubilities and locations of inoculation. Like that reported with αS PFF models [25], we show that regardless of inoculation site, pathogenic lysates from brains containing α-synucleinopathy lead to a remarkably consistent pattern of ES pathology in the *TgA53T* model. This indicates that the regional differences in the vulnerability to develop αS pathology with aging is maintained even when α-synucleinopathy is initiated by injections of pathogenic αS species. We also show that, in vivo, multiple αS species with different solubilities induce very similar αS pathologies.

Following the staging of PD pathology by Braak and colleagues [4], a series of in vitro and in vivo studies have confirmed that αS pathology can spread from an initial site of αS aggregate induction to other neurons and brain regions [5,9,10,12,13]. It is also established that lysates from murine or human tissues with αS pathology can induce early αS pathology in αS Tg mouse models [9,12]. Further, numerous studies have shown that injections of αS PFF into the CNS or peripheral tissue can induce αS pathology in both αS Tg mice and wild type mice [10,11,12,13]. While studies have generally assumed that the spreading of α-synucleinopathy occurs along the interconnected networks [5,11], it is notable that while human α-synucleinopathy may progress rostrally from the DMV, the interconnected aspect has not been demonstrated. Further, some studies using rodents show deviations from the simple cell-to-cell transmission model [25]. Thus, while the transmission of αS pathology via the interconnected network may be valid, not all neurons within the connected network are equally vulnerable to developing αS pathology. We show that progressive α-synucleinopathy is induced by injections with pathogenic αS species at different injection sites. However, the pattern of α-synucleinopathy following injections at different sites is remarkably consistent with BrSt and SpC neurons being most vulnerable to developing α-synucleinopathy. Our time course study also shows that at intermediate stages, independent of where pathogenic αS is injected, αS pathology is more prominent in BrSt and SpC neurons. These results indicate that the spread of αS pathology following pathogenic αS injections is determined, in part, by differences in neuronal vulnerability to the development of αS pathology. One potential factor could be the expression levels of endogenous αS and βS as βS is known to inhibit αS aggregation [20,21,22] and mouse αS is not efficiently templated by human αS aggregates [19].

Analysis of intermediate-stage mice for coincidence of αS pathology and glial activation indicates that αS pathology occurs before the microglial or astrocyte activation. This result agrees with a prior study demonstrating a temporal relationship between the onset of αS pathology and microglial activation following IM injections of αS PFF [27]. We extended the prior studies by showing that no obvious glial activation occurs near the αS pathology at the initial stages of pathology (Figure 4 and Figure S5). Thus, if microglia promote αS propagation [24,33], such processes are likely to occur at more advanced stages of αS pathology.

Most in vivo studies on the transmission of α-synucleinopathies have relied on αS PFF or oligomers derived from recombinant αS as the initiating factor for pathology. Although PFF can efficiently induce pathology, the biological relevance of an in vitro aggregated recombinant αS is unclear as the structure of αS PFF could be divergent from in vivo pathology. Because toxic species of αS in human PD and in vivo models, such as the *TgA53T* model, are likely to be a complex mixture of various αS species [34,35,36], characterizing the pathology induced by tissue-derived αS species will have relevance for the in vivo pathogenesis of α-synucleinopathy. This view is supported by the differential induction of pathology in huαS Tg models by various human brain-derived lysates [37]. In *TgA53T* models, αS forms soluble oligomers, particularly in the ER, which increasingly aggregate to become insoluble as the disease progresses [30,31]. This, taken together with the fact that pathogenic αS PFFs are insoluble, we initially expected that the insoluble fraction from ESL will contain most of the disease-initiating activity. Thus, we expected P150, enriched in mature pS129αS-containing aggregates, to aggressively induce αS pathology following inoculation. Surprisingly, the S150 from ES mice, despite being biochemically similar to the S150 from AS mice, is capable of inducing αS pathology in vivo. Although the exact nature of the soluble “toxic” species is unknown, our results indicate that S150 contains potentially pathogenic FILA1+ αS oligomers. Pathogenic soluble aggregates have been identified with tau pathology from the human brain [38,39], suggesting that seeding overt pathology by soluble aggregates/oligomers may be a general feature of α-synucleinopathy and tauopathy. Regardless, our results show that different species of αS with varying degrees of solubility can induce identical patterns of pathology. We also show that, consistent with the pathogenic importance of ER-associated αS pathology [30,32], microsomes from ES *TgA53T* mice are highly pathogenic in vivo. This result is consistent with prior studies showing that ER fractions from TgA53T mice are highly toxic to cultured neurons [32]. While the basis for the enhanced toxicity of ER-associated αS is under active study, the greater abundance of both FILA1+ and A11+ oligomers in the ER fraction [30] may be a contributing factor. Moreover, the interaction of αS aggregates with specific ER-associated components [40] and/or lipids [41] may also contribute to the differential pathogenicity of the ER fraction. Overall, these data indicate that further separating soluble and insoluble αS species by size and/or subcellular localization could be a fruitful approach for identifying the elusive transmissible αS species. For example, it would be significant to fractionate S150 lysate using Size Exclusion Chromatography to determine the oligomeric nature of the toxic S150 species.

In summary, our study indicates that differential neuronal vulnerability contributes to patterns of α-synucleinopathy. Thus, simple spreading of αS pathology via cell-to-cell connection cannot fully account for the progression of α-synucleinopathy. Further, our evidence supports the view that multiple αS species are capable of initiating αS pathology in vivo and it will be important to fully define the disease relevance of these different pathogenic αS species.

## 4. Materials and Methods

### 4.1. Transgenic Mouse Lines

The transgenic mouse line over-expressing A53T mutant human αS under control of the mouse prion protein promoter has been described previously (*TgA53T*, line G2-3 and line H5) [15]. *TgA53T(G2-3)* mice develop a progressive, fatal neurological disease at ~10–14 months of age and show marked αS pathology by end-stage disease. *TgA53T(H5)* lines express less αS (~50%) and do not spontaneously develop pathology. Young, asymptomatic mice aged 3–6 months were used for all experiments. Animals showing complete hindlimb paralysis were considered end-stage. All study methods were approved in full by the University of Minnesota Institutional Animal Care and Use Committee and were consistent with the National Institutes of Health Office of Laboratory Animal Welfare Policy.

### 4.2. Inoculation Material

Lysates used for all injections were generated from tissues of *TgA53T* mice at either 4 months of age (asymptomatic lysate, ASL) or end-stage (end-stage lysate, ESL) (For protocol, see 10.17504/protocols.io.4r3l22d2ql1y/v1; made available: 20 March 2024; last accessed: 27 June 2025). BrSt and spinal cord (SpC) were combined and processed together, as both regions demonstrate robust αS pathology at end-stage [15]. Preparation of the 3000× *g* lysate (S3000) has been detailed previously [9]. Briefly, affected tissue previously harvested and stored at −80 °C was sonicated in sterile saline (0.9% NaCl; 1:10 w/vol). The homogenate was centrifuged for five minutes (3000× *g*, 4 °C) and the recovered supernatant was the 3000× *g* lysate (S3000). To obtain highly soluble (S150) and insoluble (P150) fractions, S3000 fraction was centrifuged for 45 min (150,000× *g*, 4 °C). The resulting supernatant was termed the S150 fraction. The pellet was washed and resuspended in sterile saline by sonication (3x10s pulses) in half of the original volume ultracentrifuged and used as the P150 fraction.

Endoplasmic Reticulum (ER)-enriched fractions were obtained from *TgA53T* mice as previously detailed [32]. Briefly, fresh tissues were homogenized in a 1:10 (wt/vol) volume of lysis buffer (250 mM sucrose, 20mM HEPES, 10mM KCl, 1.5mM MgCl_2_, 2 mM EDTA, protease-inhibitor cocktail) and centrifuged at 1000× *g*. The supernatant was centrifuged at 10,000× *g* to pellet mitochondria and the supernatant was centrifuged at 100,000× *g* to obtain ER (pellet) and cytosol (supernatant).

Human αS preformed fibrils (PFFs) were generated from in vitro aggregation of recombinant wild type human αS monomers as previously described [16] (For protocol see: dx.doi.org/10.17504/protocols.io.81wgbxjx3lpk/v1; made available: 20 March 2024; last accessed: 27 June 2025). Human αS PFF were stored at 5 mg/mL and were sonicated and diluted to desired concentration prior to use.

### 4.3. Inoculation of TgA53T Mice

All injections into the brain were performed unilaterally into the right hemisphere/side in *TgA53T* mice at 3–6 months of age (For protocol, see dx.doi.org/10.17504/protocols.io.dm6gp3738vzp/v1; made available: 20 March 2024; last accessed: 27 June 2025). Animals were stereotaxically injected with 2.5 µg of total protein in 2.5 µL. The injections occurred at a rate of 0.1 µL per minute using a 28g needle attached via tubing to a Hamilton syringe controlled by a constant pressure syringe pump (Harvard Apparatus, Holliston, MA, USA). The needle was kept in place for five minutes post-injection. The stereotaxic coordinates used for the injection sites were as follows: intracortical/intrastriatal (IC/IS): 2.0 mm lateral from the midline, +0.2 mm relative to bregma, and 0.8 and 2.6 mm deep from the dura; BrSt: 0.2 mm lateral from the midline, −7.34 mm from bregma, 3.75 mm deep from the dura. Intramuscular (IM) injections of αS PFF were performed as described [16]. Briefly, isoflurane anesthetized mice (~3.5-months-old) were bilaterally injected into the bicep femoralis muscle bilaterally with 5 µg of PFF.

Starting at 60 days following PFF injections, the mice were evaluated for disease onset and progression three days per week (Monday, Wednesday, Friday). Disease onset was identified by an imbalance in gait leading to a wobbling phenotype and the end-stage was characterized as complete hindlimb paralysis. Animals were euthanized at either pre-determined time points or upon complete hindlimb paralysis. Half of the brain was used for immunohistochemistry and was immediately drop-fixed in 4% paraformaldehyde and remained in fixative for at least 48 h. The other half was dissected into gross brain regions, including the cortex and brainstem, and either snap-frozen on dry ice and stored at −80 °C or processed immediately. Half of the spinal cords collected remained in the spinal column and were drop-fixed in 4% paraformaldehyde and half were removed and frozen or processed in the same manner as the brains.

### 4.4. Immunohistochemistry and Mapping of αS Pathology

All fixed brains were embedded in paraffin and serially sectioned (6 µm) in the sagittal plane. Four to six sections taken every 12th section, starting at first section from midline containing hippocampus, were processed using standard immunohistochemical (IHC) techniques for DAB (Covance) detection as previously described [15,16,17] (For protocol, see dx.doi.org/10.17504/protocols.io.kxygxzqyzv8j/v1; made available: 14 December.2023; last accessed: 27 June 2025). Sections were counterstained with hematoxylin and imaged with a Leica DM2500 microscope using Leica Application Suite v4.1 imaging software (RRID: SCR_016555; https://www.leica-microsystems.com/products/microscope-software/p/leica-application-suite/, accessed on 20 March 2025). PhosphoSerine-129 αS (pS129αS)-positive inclusions/cell bodies and neurites were mapped at three lateral positions: 0.225, 1.10, and 1.525 mm from the midline using Image J v1.54e/Fiji (NIH; RRID: SCR_003070; https://imagej.net, accessed on 20 March 2025).

### 4.5. Immunofluorescence Staining

Paraffin sections of sagittal brain (BrSt-injected) and SpC (IM-injected) were processed for immunofluorescent staining as previously described [16] (For protocol, see dx.doi.org/10.17504/protocols.io.36wgq3x33lk5/v1; made available: 20 March 2024; last accessed: 27 June 2025). Briefly, the tissues were first deparaffinized followed by 30 min of antigen retrieval. Then, 3% hydrogen peroxide was used to quench endogenous peroxidases followed by blocking using 100% background sniper solution for 13 min. Sections were then incubated in primary antibodies with 5% sniper diluted in TRIS overnight at 4 °C (pS129αS, 015-25191, 1:500; Iba1, 019-19741, 1:100; Fujifilm-Wako-USA, Richmond, VA USA; GFAP, Z0334, 1:200 Dako Cytomation, Glostrup, DK) followed by incubation in secondary fluorophore-conjugated antibody (Donkey anti-mouse 488, Donkey anti-rabbit 594). Sections were imaged using a Leica Stellaris 8 confocal microscope.

### 4.6. Western Blot (Immunoblot) Analysis

Lysate preparation and immunoblot analysis were performed as previously described [16,17] (For protocol, see dx.doi.org/10.17504/protocols.io.3byl4qpqjvo5/v1; made available: 20 March 2024; last accessed: 27 June 2025). Briefly, brain tissues from mice were dissected and stored at −80 °C and frozen tissues were homogenized in TNE buffer [Tris-HCl 50 mM, NaCl 150 mM and EDTA 5 mM, and protease and phosphatase inhibitors (HALT; Thermo-Fisher; Waltham, MA, USA)]. To obtain total lysate, homogenates were solubilized with 0.5% NP40, 0.5% DOC, and 1% SDS. For TX-100 non-ionic detergent fractionation, TNE homogenates were added to 1% TX-100 and sonicated prior to centrifugation at 100,000× *g* for 20 min. Supernatant was collected as the soluble fraction while the pellet was washed and centrifuged again in 1% TX-100. The resulting pellet was then reconstituted in TNE with detergents as the TX-100 insoluble fraction [16,34].

The protein content of the lysates was determined using the BCA assay (Pierce, Thermo; Rockford, IL, USA) and samples were prepared to equal protein concentrations in reducing, SDS-sample, Laemmli buffer (Boston BioProducts; Ashland, MA, USA). For Western blot analysis, protein lysates were run on Criterion^TM^ TGX^TM^ gels (BioRad, Hercules, CA, USA) and transferred onto nitrocellulose membranes. Proteins on membranes were detected using appropriate primary antibodies followed by horseradish peroxidase (HRP)-conjugated secondary antibodies (Invitrogen, Carlsbad, CA, USA). Membranes were then developed using chemiluminescent substrates (BioRad, Hercules, CA, USA and Thermo) and imaged using the ImageQuant LAS 4000 detection system (GE Life Sciences, Piscataway, NJ, USA). Densitometry on Western blot images was analyzed using the ImageQuant TL 8.1 software (GE Life Sciences; RRID: SCR_018374; https://www.cytivalifesciences.com/en/us/shop/protein-analysis/molecular-imaging-for-proteins/imaging-software/imagequant-tl-10-2-analysis-software-p-28619, accessed on 20 March 2025).

### 4.7. Antibodies

Primary antibodies used for immunohistochemistry and immunoblot analysis are listed in Supplemental Table S1.

### 4.8. Statistical Analysis

All analyses were completed using GraphPad Prism 10 (RRID: SCR_002798; https://www.graphpad.com, accessed on 20 March 2025). Values are expressed as mean ± SD/SEM as indicated. Differences between means were analyzed by Student’s *t*-test, one-way ANOVA, or two-way ANOVA. ANOVA analyses were combined with the Tukey post hoc test for multiple comparisons (Prism 10; GraphPad Software, Boston, MA, USA). Log-rank (Mantel–Cox) test was used to assess significant differences in survival curves. An α value of 0.05 and adjusted *p* values for multiple comparisons were used to determine significance.

## Data Availability

The datasets and images used and/or analyzed, as well as a table of key resources (KRT), for the current study are available on Zenodo (https://zenodo.org/doi/10.5281/zenodo.10819209; made available: 14 March 2024; last accessed: 27 June 2025).

## Supplementary Materials

The following supporting information can be downloaded at https://www.mdpi.com/article/10.3390/ijms26136254/s1.
