# Supplementary material for: Soluble and Insoluble Lysates from the Human A53T Mutant α-Synuclein Transgenic Mouse Model Induces α-Synucleinopathy Independent of Injection Site
Source: Int J Mol Sci. 2025 Jun 28;26(13):6254. doi: 10.3390/ijms26136254 (PMC12249840; doi:10.3390/ijms26136254)
Supplement: Supplementary file 1 [file ijms-26-06254-s001.zip › ijms-3712309-supplementary.pdf]

Soluble and insoluble lysates from the human A53T mutant α-synuclein transgenic mouse model induces α-synucleinopathy independent of injection site.

Justin Barnes[1], Scott C. Vermilyea[1,2,3], Joyce Meints[1,2], Héctor Martell-Martinez[1], and Michael K. Lee[1,2,3*]

Affiliations

[1]Department of Neuroscience, University of Minnesota, Minneapolis, MN, 55455, USA

[2]Institute for Translational Neuroscience; University of Minnesota, Minneapolis, MN, 55455, USA

[3]Aligning Science Across Parkinson's (ASAP) Collaborative Research Network, Chevy Chase, MD

Supplementary Material

Table S1

Figures and Legends (Figures S1-S9)

Loading Controls	Company	Reference	RRID	Use
GAPDH (D16H11)	Cell Signaling	5174	AB_10622025	WB
α-tubulin	Abcam	4074	AB_2288001	WB
Actin	Millipore-Sigma	A2066	AB_476693	DB
**α-Synuclein Species**	**Company**	**Reference**		**Use**
α-Synuclein (total)	BD Biosciences	610787	AB_398108	WB
pS129αS	Abcam	Ab51253	AB_869973	IHC, WB
pS129αS	Wako	015-25191	AB_2537218	IF
FILA-1	Poul H. Jensen (gift)	Ref a	N/A	DB
**Glial and Neuronal Markers**	**Company**	**Reference**		**Use**
Iba1	Wako Chemical	019-19741	AB_839504	IHC, WB, IF
GFAP	Dako Cytomation	Z0334	AB_10013382	IHC, WB, IF
**Misc**	**Company**	**Reference**		**Use**
Anti-KDEL (Grp78/94)	Abcam	Ab176333	AB_2819147	WB
Grp78/Bip	Novus	NB300-520	AB_10000968	WB
CytoC	BD Biosciences	556432	AB_396416	WB
Transketolase	Invitrogen	PA5-56166	AB_2648498	WB
Calnexin	Novous	NB100-1965	AB_10002123	WB
Anti-OC (Amyloid Fibril)	Millipore-Sigma	AB2286	AB_1977024	DB

Supplementary Table S1. Antibodies used in this report. RRID-Research Resource Identifier, WB-Western Blot, DB-Dot Blot, IHC-Immunohistochemistry, IF-Immunofluorescence, N/A-not applicable.

a. Lindersson, E. *et al.* Proteasomal inhibition by alpha-synuclein filaments and oligomers. *J Biol Chem* **279**, 12924-12934 (2004).

Supplementary Figures:

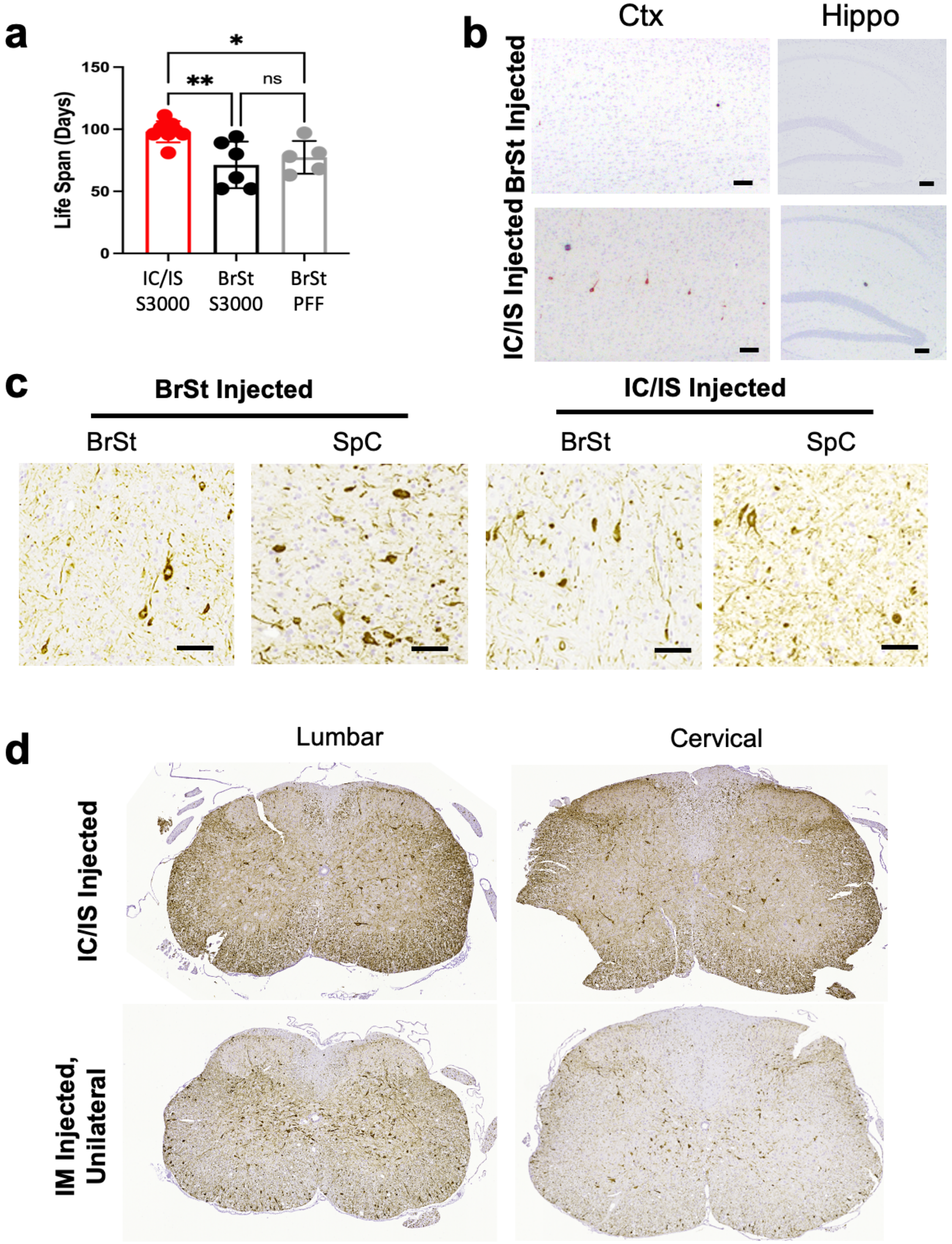

Figure S1. ***a)*** Injection of ESL or αS PFF into BrSt leads to shorter lifespan than IC/IS injections of ESL in TgA53T mice. **p<0.05*, ***p<0.01*, One-way ANOVA, *n=5-6*. ***b, c)*** αS pathology following BrSt or IC/IS injections are most prominent in BrSt and SpC at ES.

b) IC/IS injections lead to more pS129 αS+ neurons in cortex but both models lack pS129 αS in hippocampus (Hippo). ***c)*** Abundant pS129αS immunoreactivity is seen with both BrST and IC/IS injection model. ***d)*** Bilateral SpC pS129αS pathology following unilateral IC/IS injection of ESL or unilateral IM injection of αS PFF. In addition to bilateral pathology in the grey matter, pS129αS is bilaterally present in the descending and ascending axon tracts. Abbreviations: end-stage lysate, ESL; α-synuclein, αS; preformed fibril, PFF; brainstem, BrSt; intracortical/intrastriatal, IC/IS; intramuscular, IM; spinal cord, SpC; end-stage, ES. Scale Bars: 100 μm.

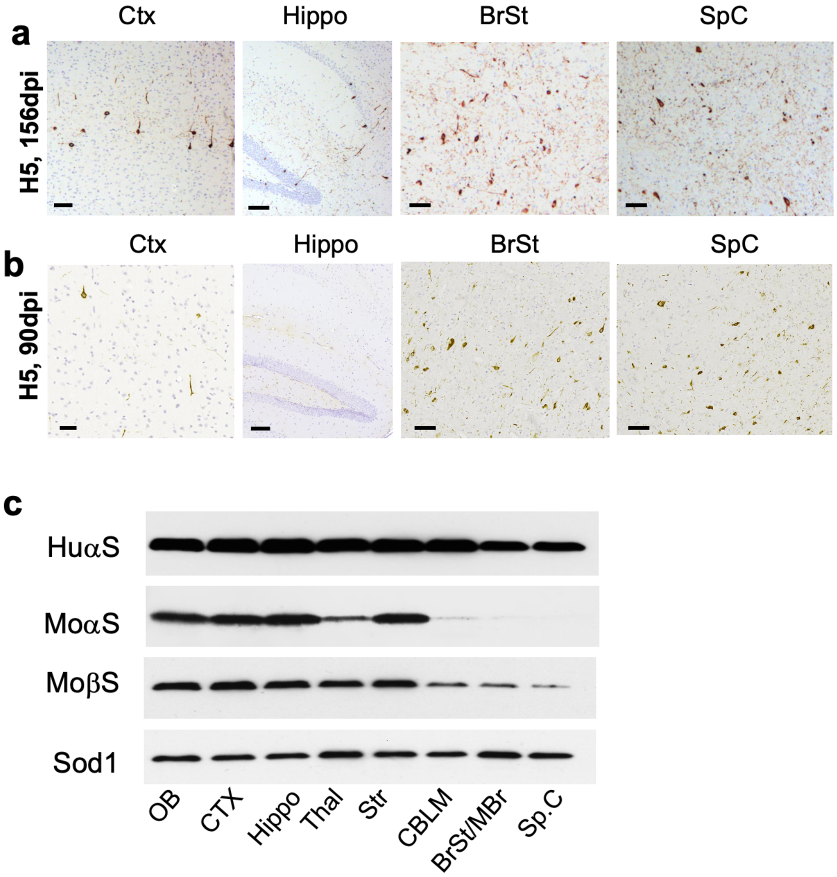

Figure S2. a, b) αS pathology following IC/IS injections of ESL into mice from *TgA53T(H5)* line. ***a)*** ES *TgA53T(H5)* mice (156 dpi) following IC/IS injection of ESL (S3000) were analyzed for accumulation of pS129 αS. While the *TgA53T(H5)* mice show more pS129 αS pathology in the cortex (CTX) and hippocampus (Hippo) than the IC/IS injected *TgA53T(G2-3)* mice (see Fig. S1B), most prominent pS129 αS pathology is localized to BrSt and SpC. ***b)*** Intermediate stage *TgA53T*(H5) (90 dpi) mice were analyzed for accumulation of pS129 αS. These mice did not show any overt motor abnormalities. There is a low level of pS129 αS staining in CTX with more abundant pS129 αS staining in the BrSt and SpC. Abbreviations: α-synuclein, αS; intracortical/intrastriatal, IC/IS; end-stage lysate, ESL; end-stage, ES. Scale Bars: 100 μm. ***c)*** Analysis of α-synuclein and β-synuclein expression in TgA53T mice. Total SDS-soluble lysates from mouse brain regions from TgA53T mice were immunoblotted using antibodies selective for human α-synuclein (HuSyn, HuαS)[28], mouse α-synuclein (moαS)[34], and βS (MoβS, Abcam). Sod1 was used to confirm equal loading. The results show that transgenic HuαS is widely expressed all brain regions. While high levels of MoαS or MoβS is expressed in forebrain areas [Olfactory Bulb (OB), Cortex (CTX), Hippocampus (Hipp), Thalamus (Thal), and Striatum (Str)] very low levels are expressed in Cerebellum (CBLM), Brain Stem/Mid-brain (BrSt/MidBr), and Spinal Cord (SpC). MoαS expression is also low in Thal.

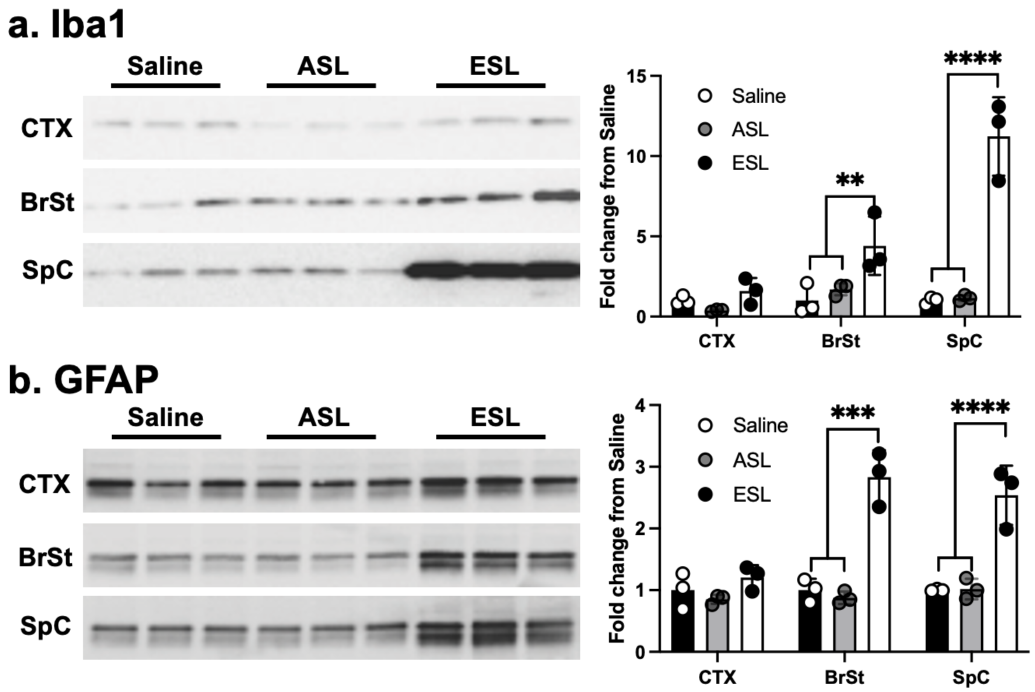

Figure S3. Quantitative analysis of increased microglia and astrocytes in ES *TgA53T* mice following ESL injection. ***a)*** Immunoblot analysis of whole-tissue lysates from CTX, BrSt, and SpC of saline-, ASL-, and ESL-injected mice for Iba-1 and corresponding quantitative analysis. Quantification of the blots confirms no change in the Iba1 signal in CTX but significant increases in BrSt and SpC (bottom). Values are mean±SD (n=3 each). Two-way ANOVA, $^{**}p<0.01$, $^{***}p<0.001$, $^{****}p<0.0001$, Tukey's *post hoc* test for multiple comparisons. n=3. ***b)*** Immunoblot analysis of lysates in ***a*** for GFAP confirms no change in GFAP levels in CTX but significant increases in BrSt and SpC (bottom). Values are mean±SD (n=3 each). One-way ANOVA, $^{*}p<0.05$. Tukey *post hoc* test for multiple comparisons. Abbreviations: end-stage, ES; end-stage lysate, ESL; cortex, CTX; brainstem, BrSt; spinal cord, SpC; asymptomatic lysate, ASL.

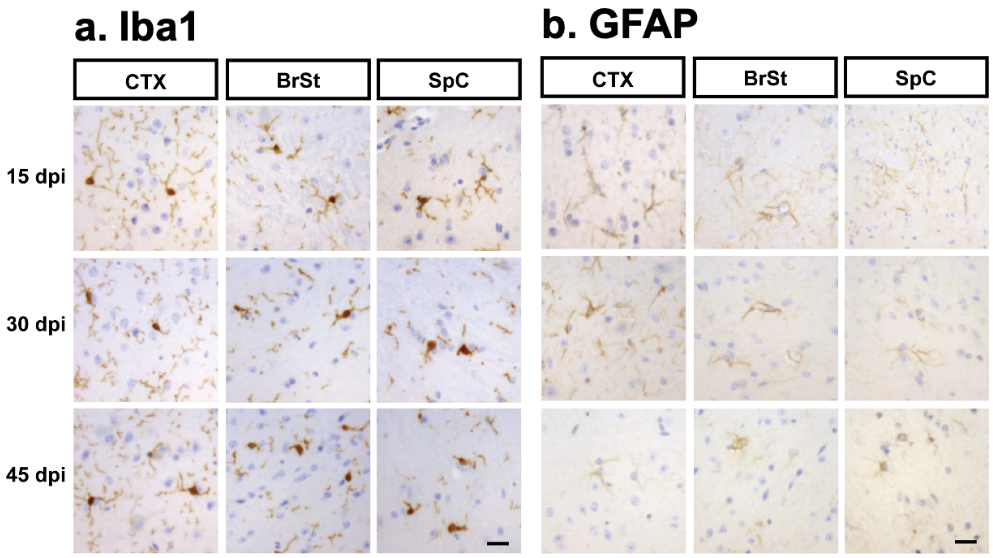

Figure S4. Tissues from end-stage lysate-BrSt injected mice were collected at 15-, 30-, and 45-days post injection and immunostained for Iba1 ***(a)*** and GFAP ***(b)***. There is no increase in neuroinflammation within 45 dpi. Increased astrogliosis and microgliosis occur only at end-stage disease when severe αS pathology is present. Abbreviations: brainstem, BrSt; cortex, CTX; spinal cord, SpC; days post-inoculation, dpi. Scale bars: 25 µm.

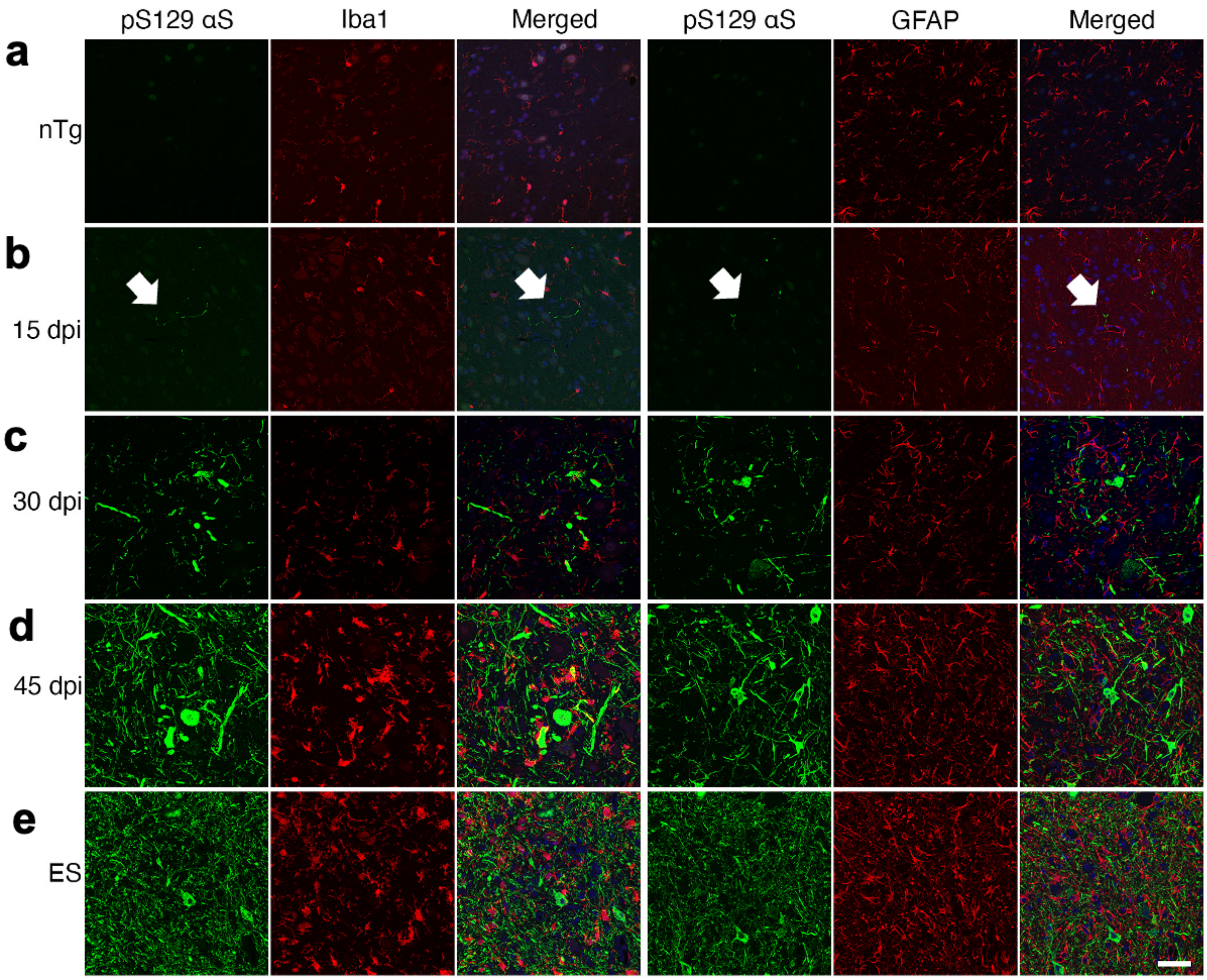

Figure S5. Neuroinflammation occurs after αS pathology in *TgA53T* mice IM injected with αS PFF. SpC sections from Control mice ***(a*, ntg)** and *TgA53T* mice injected with ESL into BrSt were analyzed at 30 dpi ***(b)*** 45 dpi ***(c)***, and ES **(*d*, injected; *e*, aged)**. The sections were stained for pS129 αS and Iba1 or GFAP. Aggregated αS (pS129 αS) is seen as early as 15 dpi ***(b, arrow)*** but the overall morphology and density of Iba-1 and GFAP staining at 15- and 30-dpi ***(b,c)*** is comparable to nTg mice ***(a)***. There are clear increases in Iba1 and GFAP staining with more established pS129 αS pathology in 45-dpi and ES animals ***(d, e)***. Abbreviations: alpha synuclein, αS; intramuscular, IM; preformed fibril, PFF; spinal cord, SpC; end-stage lysate, ESL; brainstem, BrSt; days post inoculation, dpi; non transgenic, nTg; end-stage, ES. Scale Bar: 50 μm.

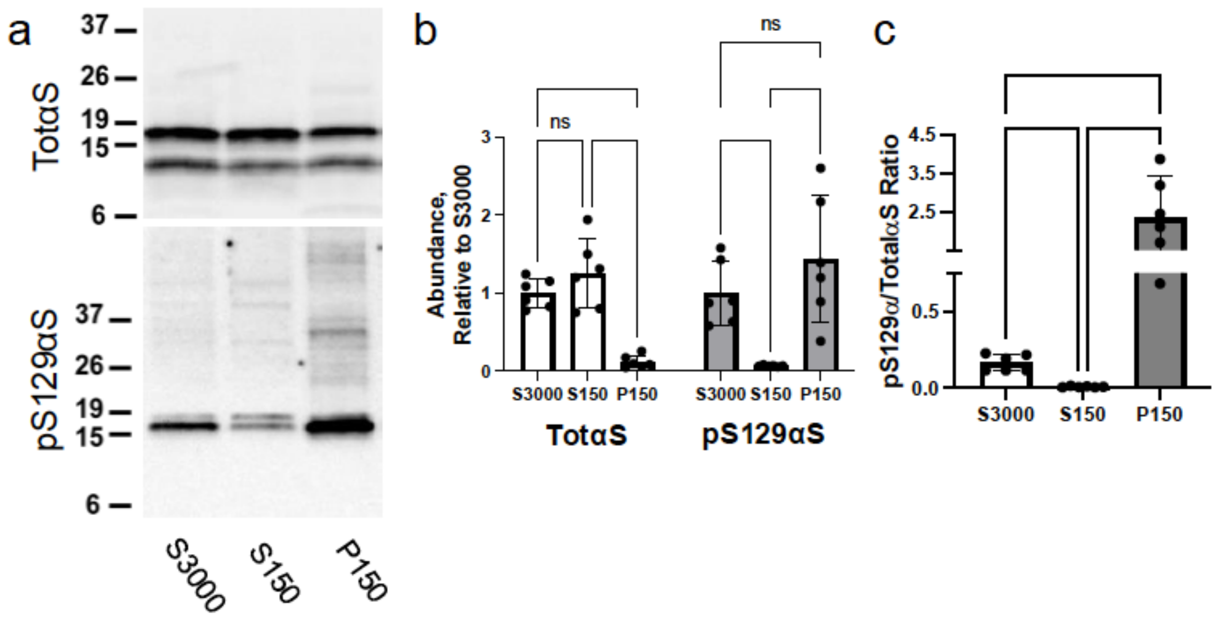

Figure S6. Immunoblot analysis of ESL, S150, and P150 injected fractions for αS. ***a)*** Immunoblot analysis of ESL (S3000), S150, and P150 fractions for total αS (TotαS) and pS129αS. 5 μg of total protein from each fraction were analyzed. TotαS enriched in S3000 and S150 while pS129αS is most abundant in P150. *b)* Quantitative analysis of TotαS and pS129αS in S3000, S150, and P150. The abundance was normalized to the average levels in S3000. n=6 animals per group; ***p<0.01, ***p<0.001, ****p<0.0001*; Two-way ANOVA, Tukey's *post hoc* test for multiple comparisons. c) Relative abundance of pS129αS normalized to TotαS. n=6 animals per group; **p<0.05, **p<0.01*, One-way ANOVA, Tukey's *post hoc* test for multiple comparisons.

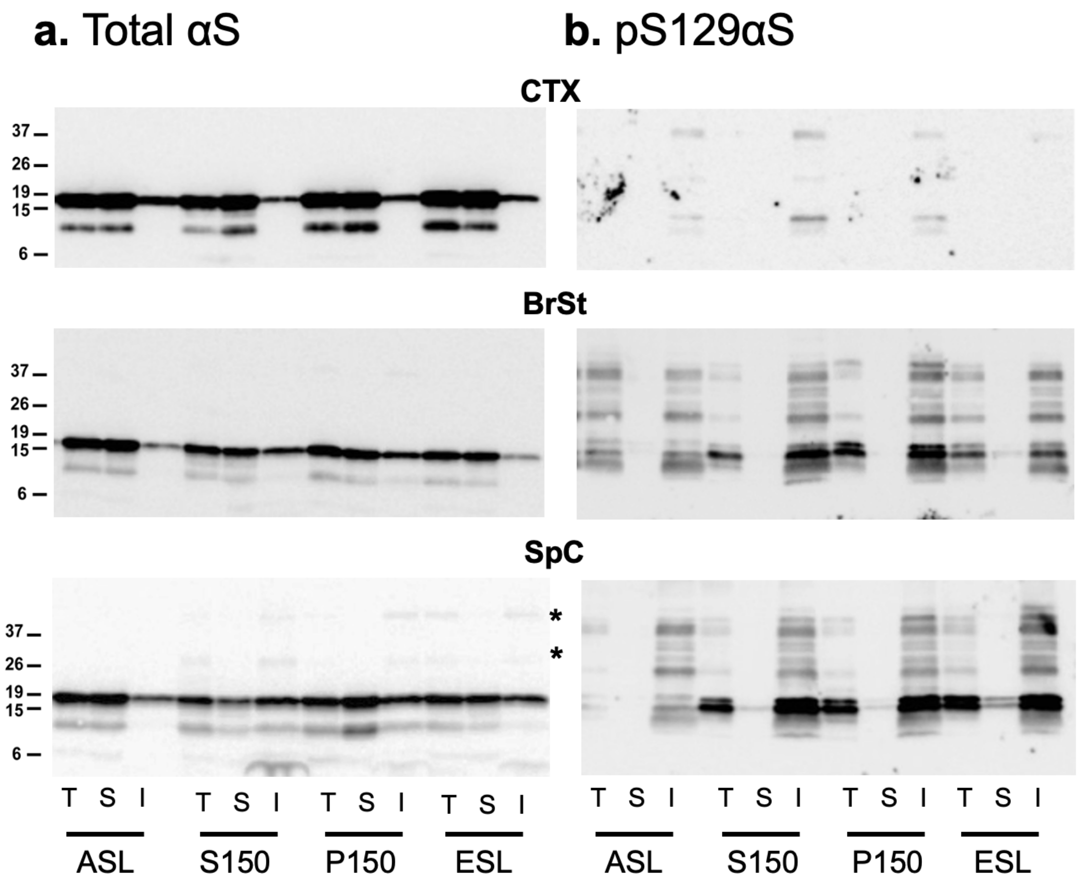

Figure S7. Immunoblot analysis of αS in ASL, ESL, S150, and P150 injected *TgA53T* mice. Whole tissue lysates (T) from CTX, BrSt, and SpC were further fractionated into 1% Tx-100 soluble (S) and insoluble (I) fractions. Equal amounts of proteins were analyzed for Total αS ***(a)*** and pS129 αS ***(b)***. Virtually all the pS129 αS accumulates in the insoluble fractions of BrSt and SpC, along with HMW αS (*). While the highest levels of total αS is expressed in the CTX, the level of pS129 αS is minimal and do not increase with injection of pathogenic lysates. No signs of αS aggregation is seen with the ASL. Abbreviations: α-synuclein, αS; asymptomatic lysate, ASL; end-stage lysate, ESL; cortex, CTX; brainstem, BrSt; spinal cord, SpC; high molecular weight, HMW.

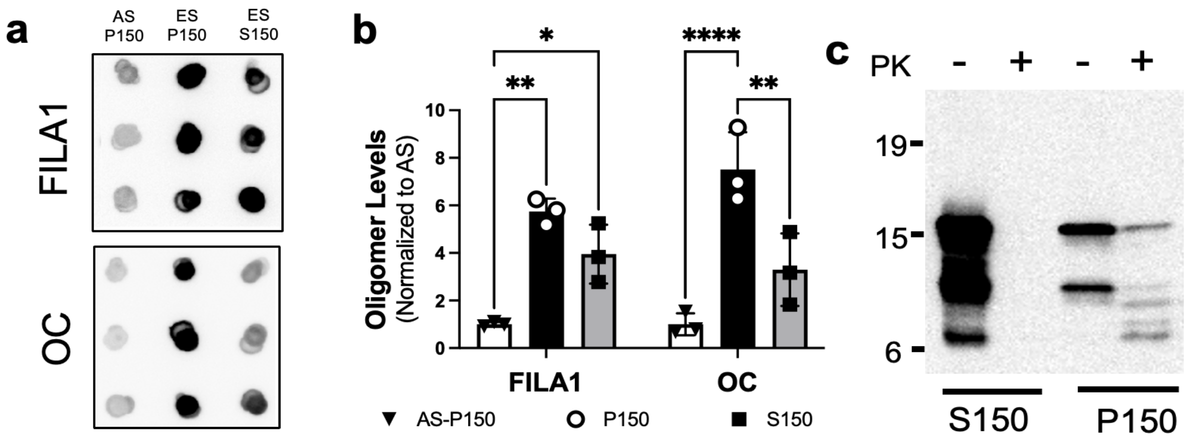

Figure S8. Analysis of αS oligomers in S150 and P150 fractions. ***a)*** Dot blot analysis of S150 and P150 for FILA1 and OC immunoreactivity. Equal amount of total protein (2 μg) from each fraction were dotted on to nitrocellulose membrane and probed with FILA1 antibody and OC antibody. ***b)*** Quantitative analysis of dot blots shown in ***a***. Levels of immunoreactivity was normalized to AS-P150. In P150, both FILA1 and OC immunoreactivity are higher than in AS-P150. In S150, FILA1 levels are higher but the OC levels are not different than in AS-150. n=3, *$^{*}p<0.05$, $^{**}p<0.01$, $^{****}p<0.0001$*, One-way ANOVA, Tukey's *post hoc* test for multiple comparisons. ***c)*** S150 and P150 containing equal amount of protein (5 μg) were incubated with 50 μg/ml proteinase K (PK) and 1% Triton x-100 for 20 min on ice. The reaction was stopped by the addition of 2mM (final concentration) of phenylmethylsulfonyl fluoride. The fractions were immunoblotted for total αS. Virtually all the αS in S150 is degraded by PK while both full length and partially proteolyzed αS survive PK treatment in P150. Abbreviations: alpha-synuclein, αS; asymptomatic, AS; end-stage, ES.

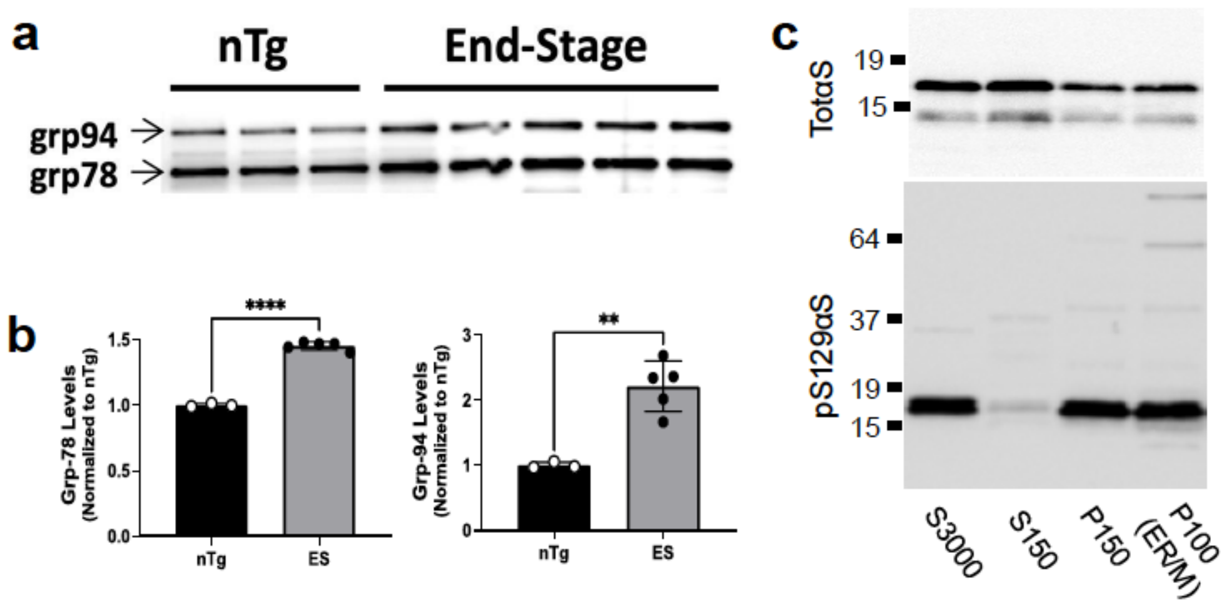

Figure S9. Increased ER chaperones in ES mice injected with ESL into BrSt. ***a)*** Whole SpC lysates from nTg and End-Stage *TgA53T* mice injected with ESL were immunoblotted using anti-KDEL antibody (2D6, Invitrogen) that recognizes both Grp78 and Grp94. ***b)*** Quantitative analysis of ***a*** shows that both ER chaperones are increased in TgA53T mice. **$p<0.01$, ****$p<0.0001$, unpaired *t*-test, two-tailed. nTg, n=3; Tg-End Stage, n=5. ***c)*** Immunoblot comparison of total αS and pS129αS in end-stage lysates and fractions [S3000, S150, P150, and P100(ER/M)]. Equal amount of total protein (5 μg) from each fraction were analyzed. Despite the lower levels of total αS, pS129αS is enriched in P150 and P100. Abbreviations: endoplasmic reticulum, ER; end-stage, ES; end-stage lysate, ESL; nontransgenic, nTg; transgenic, Tg.
